# Ultrasonographic Estimation of Total Brain Volume: 3D Reliability and 2D Estimation. Enabling Routine Estimation During NICU Admission in the Preterm Infant

**DOI:** 10.3389/fped.2021.708396

**Published:** 2021-07-22

**Authors:** Isabel Benavente-Fernández, Estefanía Ruiz-González, Manuel Lubian-Gutiérrez, Simón Pedro Lubián-Fernández, Yunior Cabrales Fontela, Cristina Roca-Cornejo, Pedro Olmo-Duran, Simón Pedro Lubián-López

**Affiliations:** ^1^Division of Neonatology, Department of Paediatrics, Puerta del Mar University Hospital, Cádiz, Spain; ^2^Biomedical Research and Innovation Institute of Cádiz (INiBICA) Research Unit, Puerta del Mar University, Cádiz, Spain; ^3^Area of Paediatrics, Department of Child and Mother Health and Radiology, Medical School, University of Cádiz, Cádiz, Spain; ^4^Department of Paediatrics, Puerta del Mar University Hospital, Cádiz, Spain

**Keywords:** 3D ultrasonography, preterm infant, lineal measure, brain volume, magnetic resonance imaging

## Abstract

**Objectives:** The aim of this study is to explore if manually segmented total brain volume (TBV) from 3D ultrasonography (US) is comparable to TBV estimated by magnetic resonance imaging (MRI). We then wanted to test 2D based TBV estimation obtained through three linear axes which would enable monitoring brain growth in the preterm infant during admission.

**Methods:** We included very low birth weight preterm infants admitted to our neonatal intensive care unit (NICU) with normal neuroimaging findings. We measured biparietal diameter, anteroposterior axis, vertical axis from US and MRI and TBV from both MRI and 3D US. We calculated intra- and interobserver agreement within and between techniques using the intraclass correlation coefficient and Bland-Altman methodology. We then developed a multilevel prediction model of TBV based on linear measurements from both US and MRI, compared them and explored how they changed with increasing age. The multilevel prediction model for TBV from linear measures was tested for internal and external validity and we developed a reference table for ease of prediction of TBV.

**Results:** We used measurements obtained from 426 US and 93 MRI scans from 118 patients. We found good intra- and interobserver agreement for all the measurements. US measurements were reliable when compared to MRI, including TBV which achieved excellent agreement with that of MRI [ICC of 0.98 (95% CI 0.96–0.99)]. TBV estimated through 2D measurements of biparietal diameter, anteroposterior axis, and vertical axis was comparable among both techniques. We estimated the population 95% confidence interval for the mean values of biparietal diameter, anteroposterior axis, vertical axis, and total brain volume by post-menstrual age. A TBV prediction table based on the three axes is proposed to enable easy implementation of TBV estimation in routine 2D US during admission in the NICU.

**Conclusions:** US measurements of biparietal diameter, vertical axis, and anteroposterior axis are reliable. TBV segmented through 3D US is comparable to MRI estimated TBV. 2D US accurate estimation of TBV is possible through biparietal diameter, vertical, and anteroposterior axes.

## Introduction

Very low birth weight infants (VLBWI) are a population at high risk for cognitive, motor, neurosensory, and behavioral disability (1). These sequelae are associated with findings of brain injury and/or impaired growth of different brain structures (2).

Routine neonatal brain imaging via ultrasound (US) and magnetic resonance imaging (MRI) are usually evaluated through visual qualitative assessment of the images which leads to loss of information and variability among observers (3–6). To improve reproducibility in assessment of brain growth, composite and global scores have been developed that include a combination of subjective items and objective measures. These composite scores incorporate quantitative measurements of different structures such as corpus callosum thickness, lateral ventricles width, biparietal diameter, cerebellar height and diameter, subarachnoid space dimensions, and interhemispheric distance (7–9). While these scores are undoubtedly useful for the neonatologist, an approach to US total brain volume estimation could provide new useful information.

A global reduction in brain volume can be visualized through these neuroimaging techniques and, when severe, it has been shown to be associated with adverse neurodevelopmental outcome (10). However, a more subtle tissue loss can be overlooked and therefore prognosis information can be inaccurate. This tissue loss could be hypothesized to occur in the context of brain dysmaturation, an increasingly recognized problem in the preterm infant that leads to impaired myelination and delayed cortical development (11). Despite survival of the extreme preterm infant without an increase in the prevalence of severe brain injury, the long-term outcome is still compromised in up to 70% of preterm infants. A better approach to the evaluation of brain growth pattern during admission in the NICU could help in the identification of this dysmaturation process. While some researchers have proposed different linear measurements as a proxy of early measurement of brain volume (12, 13), these studies have not compared the estimated TBV with manual segmentation in 3D US or MRI estimated TBV, which could be considered the gold standard. Moreover, longitudinal estimation of brain volume though US has not been systematically tested as most of the studies rely on MRI for volumetric assessment of brain structures.

3D US allows an acquisition of the whole brain and navigation in three orthogonal planes which can improve orientation and symmetry of two-dimensional views (14). Furthermore, it enables a volumetric approach of the brain, which could lead to longitudinal assessment of brain growth during the neonatal period and facilitate impact of neonatal comorbidities on early brain growth.

The aim of this study is to determine the reproducibility of total brain volume estimated through 3D US and the accuracy of this estimation when compared to the gold standard MRI technique.

As 2D US is the standard of care we also wanted to analyse if three axes, one in each orthogonal plane of the brain could be a good estimate of TBV both in MRI and 3D US. We hypothesize that, if feasible, it could be implemented in the routine US evaluation during admission.

## Patients and Methods

### Patients

This study is part of a longitudinal cohort that includes VLBWI born at Hospital Puerta del Mar, Cádiz, Spain as of May 2018 with recruitment still ongoing. The study (PI0052/2017) aims to investigate the association of pre- and perinatal factors, brain growth and brain injury and socioeconomic status with long term neurodevelopmental outcome in the preterm infant. We consecutively enrolled VLBWI who met inclusion criteria (weight at birth equal or <1,500 grams, gestational age at birth equal or <32 weeks of gestation) and whose parents or legal guardians had signed informed consent. Exclusion criteria consisted of congenital and chromosomal anomalies, metabolic disorders and central nervous system infections. For the purpose of this study, we included those who were born from May 2018 to December 2019. We further excluded those with abnormal brain US or MRI findings (any degree of germinal-matrix/intraventricular hemorrhage and/or white matter injury). Perinatal data and details of the infants' clinical course were prospectively collected. All patients were followed prospectively and underwent weekly cranial US until either discharge or term-equivalent age. We strived to perform two MRIs: one early scan, done as soon as the patient was clinically stable and another one at term equivalent age. The same day the MRI was done we performed a 3D US as per protocol to enable comparison of both neuroimaging tools.

### Brain MRI

MRI scans were performed using 1.5 T scanner (Magneton Symphony, Siemmens Health Care, Erlangen, Germany) located in the radiology unit. T1-weighted images were obtained using a three-dimensional (3D) spoiled gradient [repetition time 1,660 (TR)/echo time 5.16(TE)] and transverse T2-weighted turbo spin-echo imaging (4,180.00/98.00). MRI measures were obtained using ITK-SNAP (http://www.itksnap.org/) segmentation tool (15).

### 2D and 3D US

Weekly cranial 2D US and 3D US were performed with the infant lying supine with his or her head turned to the right. Volume acquisition was carried out through the 4D option in the 3D/4D Voluson i portable ultrasound system (GE Healthcare) as previously described by our group (14, 16). Through this option, with the transducer positioned in the third coronal plane, the beam moves from anterior to posterior planes using a center frequency of 6.5 MHz with a scan angle set at 90°. Scans were saved and analysis was performed off-line by using 4D View software (version 17.0; GE Healthcare).

### US and MRI Measurements

The same linear measurements were performed in MRI and US searching for the most similar plane in both techniques following the same anatomical landmarks. Parenchymal biparietal diameter (BPD), anteroposterior (AP) axis and vertical axis were measured in millimeters, and total brain volume (TBV) was measured in cubic centimeters.

**Figure 1:**
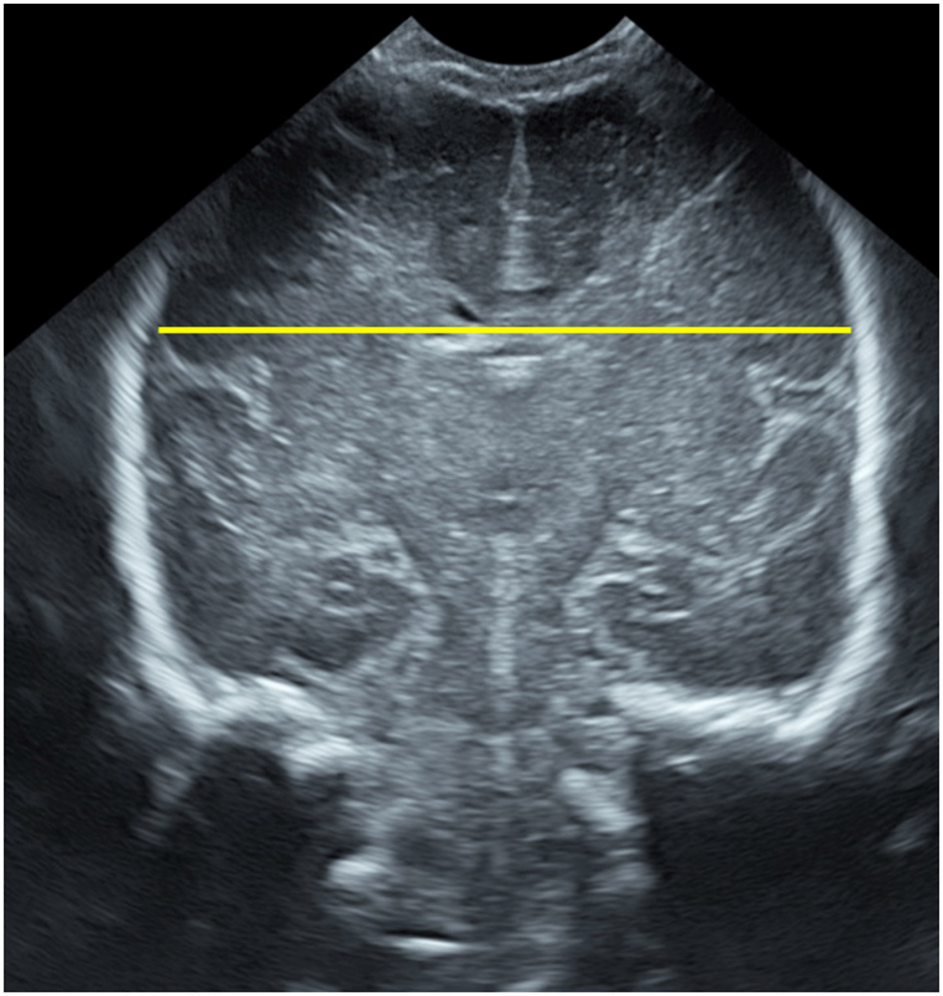
Biparietal diameter measured on the ultrasonographic third coronal plane.

BPD has been extensively studied and we have applied the same anatomical landmarks measuring, in the 3rd coronal plane the maximal distance from side to side of parietal cortex (9) (see Figure 1).

**Figure 2:**
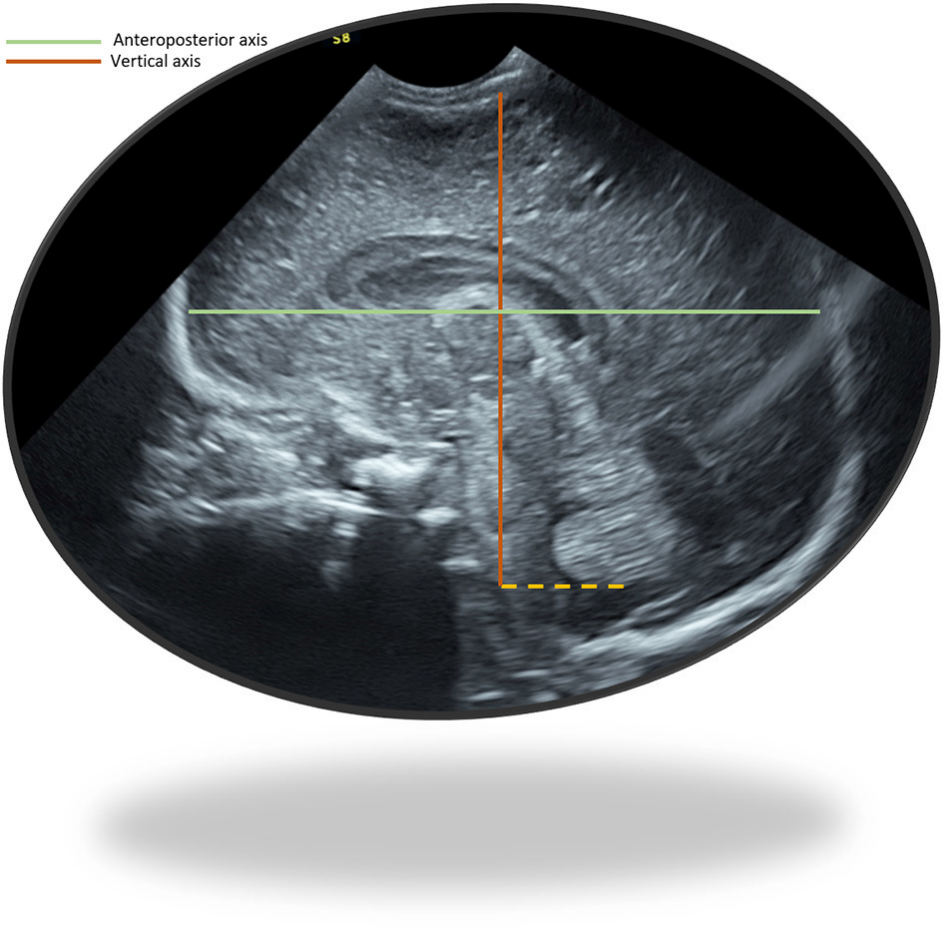
Anteroposterior axis and vertical axis measured in an ultrasonographic sagittal plane.

The anteroposterior and vertical axes were newly defined for this study, trying to make them easy and reliable, to diminish variability among observers. A mid-line sagittal view was obtained ensuring the contours of the vermis were clearly seen, with an orientation enabling the anterior vermis contour to be followed as an imaginary vertical line. A horizontal line was then drawn at the level of the inferior vermis contour. This line was considered to be the inferior limit of the vertical axis. The vertical axis was then drawn, starting at the cerebral cortex, visible just below the transducer as a narrow echogenic line, bordered by a lower echogenicity and broader line, and representing the maximum distance between the cerebral cortex and the inferior limit. The anteroposterior axis was always drawn after the vertical axis was defined to ensure a 90 degrees angle among both axes and was the maximum horizontal distance from the frontal to the occipital cortex. When defining the cortex was difficult, the inner bony mantel was used (Figure 2).

3D US TBV estimation was performed through manual segmentation using the Virtual Organ Computer-Aided Analysis (VOCAL) method (GE Healthcare) which allows manual contouring of consecutive 2D US planes with a 30° rotation angle and a final 3D renderization (14, 17).

### Statistical Analysis

The intra- and interobserver reliabilities were assessed by two observers (S.L.F. and M.L.G. for MRI scans and I.B.F and E.R.G for US scans) blinded to clinical information. Based on previous studies by our group (16), we estimated a necessary sample size ranging from 12 to 22 to detect relevant differences for different measures within and among both techniques. All linear measurements were repeated three times to evaluate intraobserver variability. To control for memory effects potentially biasing subsequent measurements, each observer performed all the measurements for all patients prior to starting again with the first one, with a 24-h interval between repeat measurements of the same subject and recorded them while blinded to previous measurements. The interobserver reliability was evaluated by comparing the mean of the three measures performed by each observer.

The intraclass correlation coefficients were calculated by using the two way random model for absolute agreement and interpreted according to the strength of agreement scale by Brennan and Silman (18). Very poor agreement is considered if the ICC <0.4, poor if the ICC approaches 0.4, good if the ICC approaches 0.75 and excellent if the ICC >0.75.

Furthermore, Bland-Altman (BA) analysis was also used as it allows a graphical representation of reproducibility and adds value through a complementary quantitative analysis (19).

We estimated the linear and TBV measurements by post-menstrual age expressing the mean and the population 95% confidence interval values of the mean.

As 3D US is not a standard US tool available in most NICUs, we aimed to facilitate volumetric estimation of TBV based on 2D US measurements. Using mixed effects regression models we compared the prediction of TBV based on linear measurements of the three brain axes made from 3D US and from MRI.

After model estimation, its reliability was further assessed through internal and external validation. External validity of the model was tested by evaluating its loss of prediction (shrinkage) after randomly splitting the sample into a training group and validation group. The model is considered acceptable if the shrinkage is <10%. Internal validity of the model was performed through cross-validation. This procedure splits the data randomly into *k* partitions, then for each partition it fits the specified model using the other *k*−1 groups and uses the resulting parameters to predict the dependent variable in the unused group. Finally, crossfold reports a measure of goodness-of-fit from each attempt and the mean value for all (*R*${}_{\text{Mean}}^{2}$) which is interpreted as the real predictive ability of the model when performed on external data.

Statistical analysis was conducted using Stata 16.0 (Stata Statistical Software: Release 16. College Station, TX: StataCorp LP).

## Results

### Study Population

During the study period 156 VLBW were admitted to the NICU at Hospital Puerta del Mar in Cádiz, Spain. We excluded 38 patients with abnormal US findings such as germinal-matrix/intraventricular hemorrhage and/or white matter injury. US linear measurement of the three orthogonal axis and 3D US brain volume manual segmentation was performed in each US of all 118 patients, with 426 US measured with a median of 4 US per patient [IQR 2–7].

A brief description of the main perinatal characteristics of the included patients is given in Table 1.

**Table 1:** Perinatal characteristics of the included patients (*n* = 118).

**Gestational age (weeks)**	**30 [28–31]**
**Birth weight (grams)**	**1,175 [850–1,465]**
**Apgar 1 min**	**6 [5–7]**
**Apgar 5 min**	**8 [7–9]**
**Sex (female)**	**59 (50%)**
**Small for gestational age**	**21 (17,8%)**

*Quantitative variables are expressed as median interquartile range [IQR]. Small for gestational age (SGA) was considered when birth weight was <10th percentile for gestational age according to Intergrowth-21st curves (20)*.

The same measurements were performed in 93 MRIs that were available from 62 patients (31 patients had 2 MRIs). For the reliability analysis we compared US to MRI measurements of 21 randomly selected patients who had their US scans performed within a ±24 h interval from the MRI. For the prediction model of MRI estimation of TBV though the linear measurements we used all the measurements performed in the 92 MRI scans.

### Total Brain Volume

TBV 3D segmentation obtained excellent intra- and interobserver reliability indices both in 3D US and in MRI, as measured by ICC which ranged from 0.99 (95% CI 0.95–1.00) to 0.99 (95% CI 0.99–0.99). When comparing 3D US TBV segmentation to MRI TBV we obtained an ICC of 0.98 (95% CI 0.96–0.99). Moreover, using Bland-Altman method and plotting both US and MRI measurements (see Tab “TBV” in Supplementary Material) we could see that US measure of total brain volume is reliable compared to MRI TBV measure with a non-significant mean difference of 10.16 cm^3^ (95% CI −0.38–20.7).

**Figure 3:**
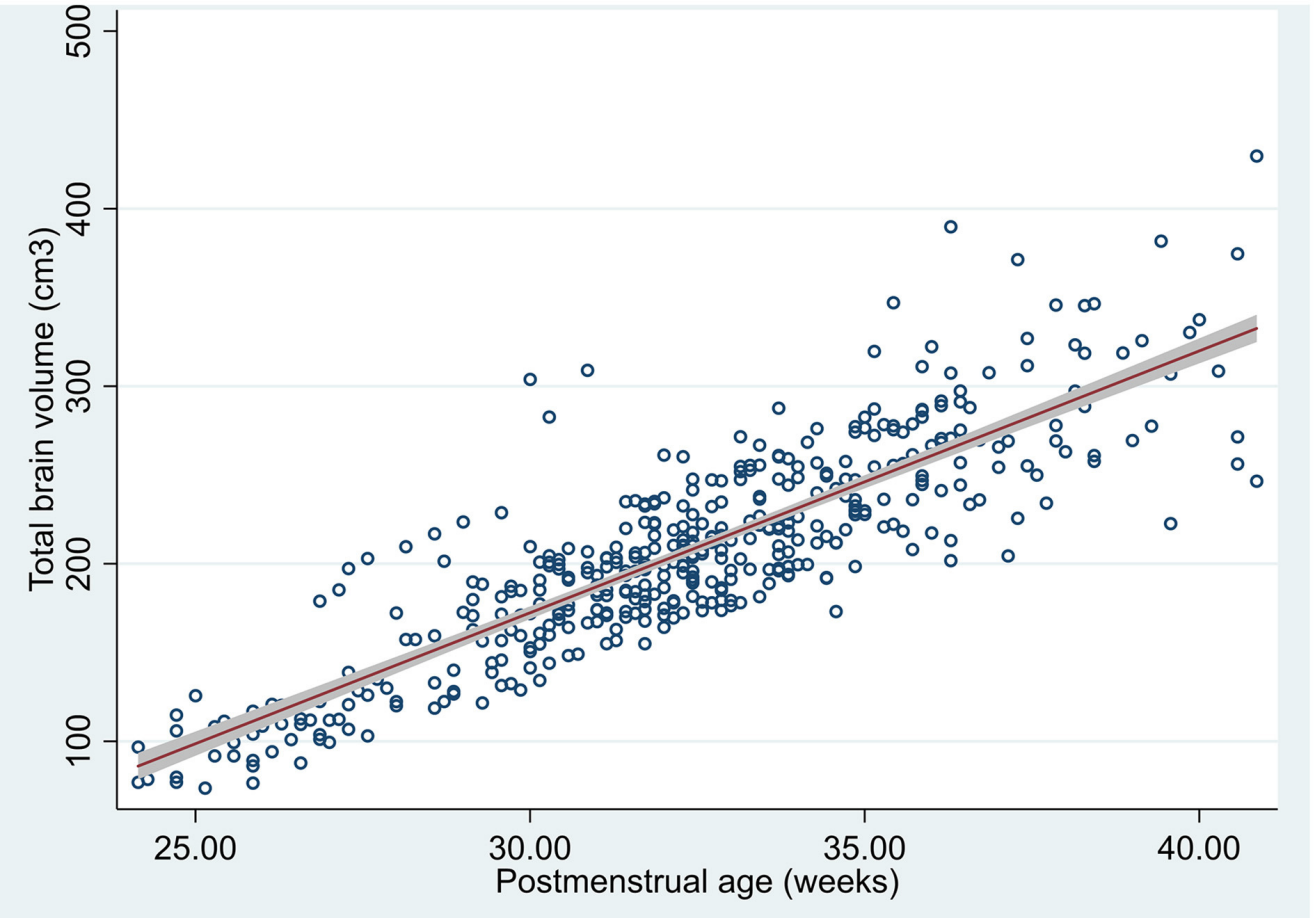
Scatterplot of total brain volume by post-menstrual age segmented in 3D US, predicted mean and 95% confidence interval of the population means.

We estimated the means in the measured US and 95% confidence interval in our population means of TBV manually segmented in the 426 3D US by post-menstrual age (Table 2 and Figure 3).

**Table 2:** Observed means and 95% confidence interval for population means of US total brain volume (cm^3^) by post-menstrual age.

**PMA (weeks)**	***N***	**Mean TBV (sd)(observed)**	**95% CI for population means**
24	8	89.64 (14.63)	77.41–101.87
25	12	97.97 (15.95)	87.83–108.10
26	17	114.49 (19.63)	104.40–124.58
27	15	135.88 (33.31)	117.45–154.33
28	16	152.12 (32.78)	134.65–169.59
29	25	167.12 (27.03)	155.97–178.28
30	39	186.78 (39.07)	174.11–199.44
31	55	193.88 (22.18)	187.89–199.88
32	62	204.81 (23.69)	198.80–210.83
33	47	221.96 (28.46)	213.61–230.32
34	34	231.58 (26.16)	222.45–240.71
35	30	263.56 (31.87)	251.67–275.46
36	24	271.37 (40.18)	254.40–288.33
37	15	276.16 (45.53)	250.95–301.37
38	11	302.06 (31.98)	280.57–323.54
39	8	306.03 (46.66)	265.35–346.71
40	8	319.06 (62.47)	266.84–371.29

### Biparietal Diameter

Parenchymal BPD measurements obtained excellent intra- and interobserver reliability indices both in 3D US and in MRI, as measured by ICC which ranged from 0.86 (95% CI 0.60–0.95) to 0.99 (95% CI 0.96–0.99).

When comparing US BPD estimation to MRI BPD, we obtained an ICC of 0.84 (95% CI 0.51–0.94). Using Bland-Altman method and plotting both US and MRI measurements (see Tab “BPD” in Supplementary Material) we could see that US measure of BPD overlaps MRI BPD measure with a non-relevant mean difference of −2.1 (95% CI −3.7–0.61).

**Figure 4:**
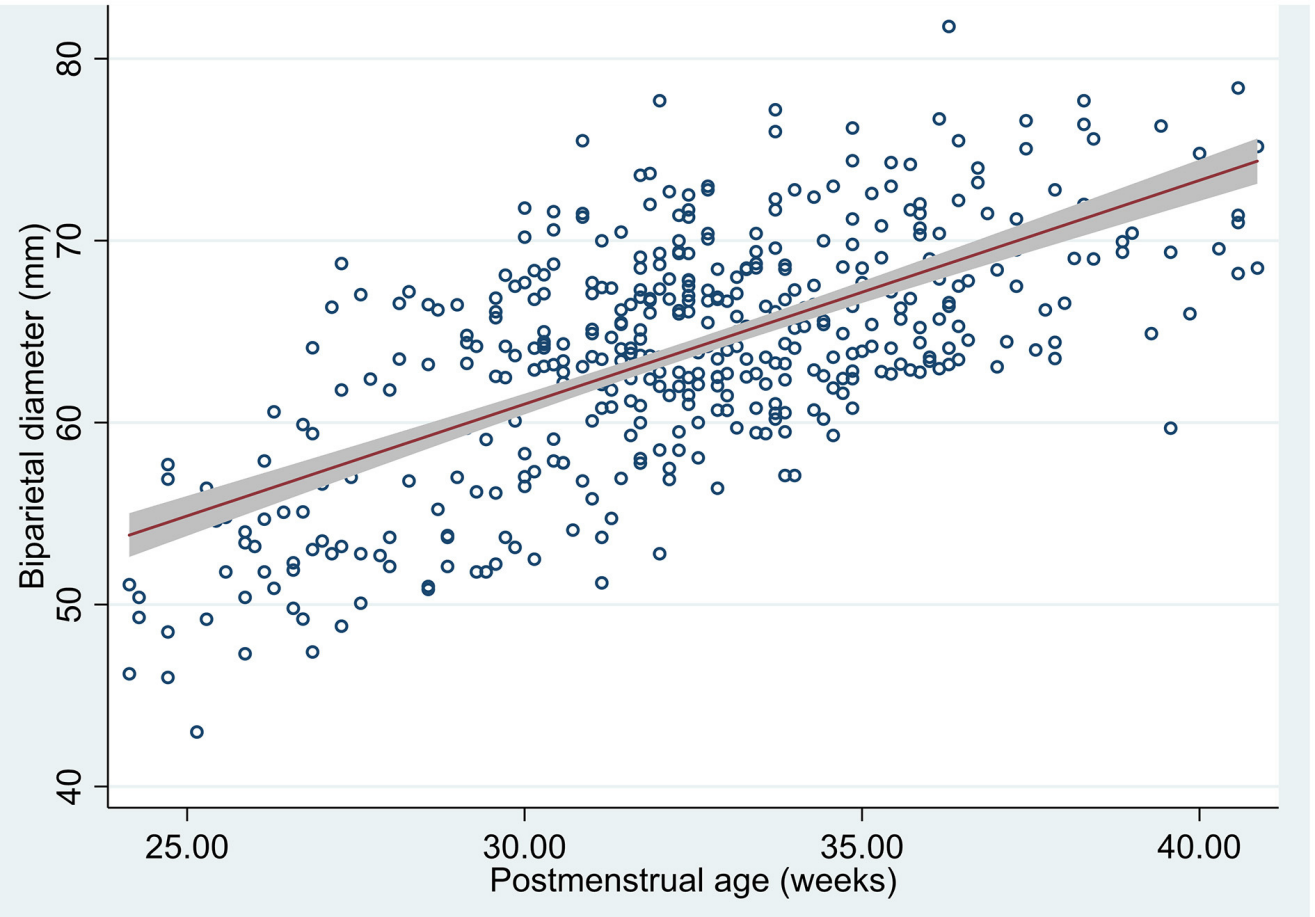
Scatterplot of biparietal diameter by post-menstrual age measured in US, predicted mean and 95% confidence interval of the population means.

We estimated the means in the measured US (425 BPD measurements were obtained of the 426 US) and 95% confidence interval in our population means of BPD by post-menstrual age (Table 3 and Figure 4).

**Table 3:** Mean US biparietal diameter (sd) in millimeters per week of post-menstrual age and 95% CI for population means.

**PMA (weeks)**	***N***	**Mean BPD (sd)(observed)**	**95% CI for population means**
24	8	50.76 (4.42)	47.07–54.46
25	12	52.13 (4.00)	49.58–54.67
26	17	54.49 (4.55)	52.15–56.83
27	15	57.46 (6.39)	53.93–61.00
28	16	58.39 (6.33)	55.02–61.76
29	25	60.85 (5.40)	58.62–63.08
30	39	63.74 (5.50)	61.96–65.52
31	55	63.83 (4.58)	62.59–65.07
32	62	65.30 (4.85)	64.07–65.53
33	47	65.10 (4.40)	63.81–66.39
34	34	65.74 (4.61)	64.13–67.35
35	30	67.61 (3.72)	66.23–69.00
36	24	68.54 (4.97)	66.44–70.64
37	15	68.44 (4.25)	66.08–70.79
38	10	71.69 (3.69)	69.05–74.33
39	8	68.57 (5.08)	64.32–72.82
40	8	72.13 (3.64)	69.09–75.17

### Anteroposterior Axis

Anteroposterior axis measurements obtained excellent intra- and interobserver reliability indices both in 3D US and in MRI, as measured by ICC which ranged from 0.94 (95% CI 0.56–0.98) to 0.99 (95% CI 0.99–1.0). When comparing US anteroposterior axis estimation to MRI, we obtained an ICC of 0.83 (95% CI 0.07–0.95) (see Supplementary Material). Using Bland-Altman method and plotting both US and MRI measurements (see Tab “AP axis” in Supplementary Material) we could see that US measure of anteroposterior axis overlaps MRI measured anteroposterior axis with a non-relevant mean difference of 3.62 (95% CI 2.14–5.1).

**Figure 5:**
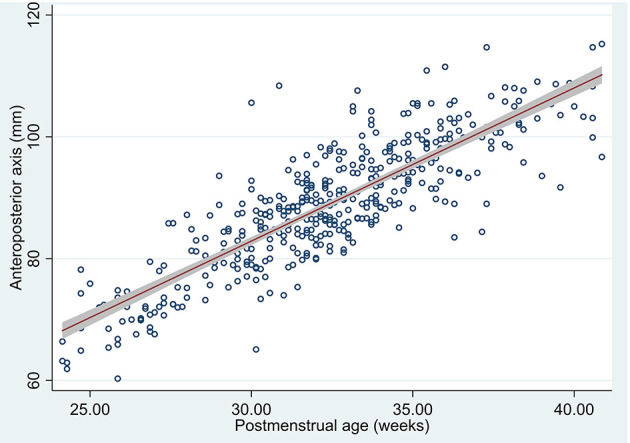
Scatterplot of anteroposterior axis by post-menstrual age measured in US, predicted mean and 95% confidence interval of the population means.

We estimated the means in the measured US (423/426 anteroposterior axis measurements were obtained) and 95% confidence interval in our population means of anteroposterior axis by post-menstrual age (Table 4 and Figure 5).

**Table 4:** Mean US anteroposterior axis (sd) in millimeters per week of post-menstrual age and 95% CI for population means.

**PMA (weeks)**	***N***	**Mean AP axis (sd)(observed)**	**95% CI for population means**
24	8	67.55 (5.87)	62.65–72.45
25	11	69.77 (4.78)	66.56–72.98
26	17	71.54 (2.93)	70.03–73.04
27	15	74.72 (5.28)	71.80–77.64
28	16	80.08 (4.39)	77.74–82.41
29	25	82.20 (4.41)	80.68–84.32
30	39	84.75 (7.74)	82.24–87.26
31	54	87.15 (5.07)	85.76–88.53
32	62	88.77 (5.03)	87.49–90.05
33	47	92.54 (6.39)	90.66–94.42
34	34	94.49 (4.51)	92.91–96.06
35	30	98.06 (5.24)	96.10–100.02
36	23	98.57 (6.33)	90.66–94.42
37	15	100.18 (7.41)	96.07–104.28
38	11	103.48 (4.14)	100.69–106.26
39	8	102.64 (6.50)	97.21–108.07
40	8	105.78 (6.62)	100.25–111.31

### Vertical Axis

Vertical axis measurements obtained excellent intra- and interobserver reliability indices both in 3D US and in MRI, as measured by ICC which ranged from 0.87 (95% CI 0.23–0.96) to 0.99 (95% CI 0.99–0.99).

When comparing US vertical axis estimation to MRI vertical axis we obtained an ICC of 0.75 (95% CI 0.34–0.90). Using Bland-Altman method and plotting both US and MRI measurements (see Tab “Vertical axis” in Supplementary Material) we could see that US measure of vertical axis overlaps MRI vertical axis measure with a non-relevant mean difference of 1.7 (95% CI 0.44–3.01).

**Figure 6:**
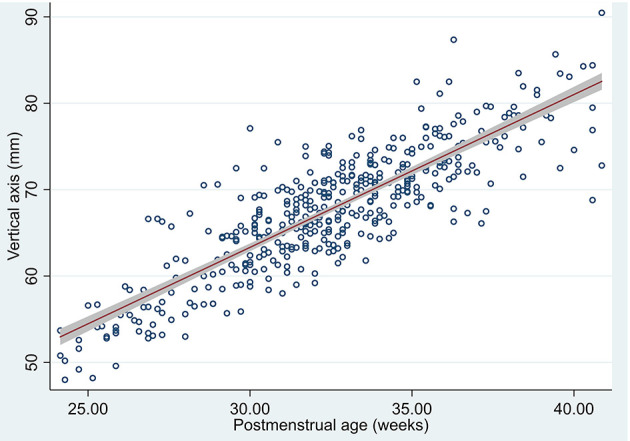
Scatterplot of vertical axis by post-menstrual age measured in US, predicted mean and 95% confidence interval of the population means.

We estimated the means in the measured US (426/426 vertical axis measurements were obtained) and 95% confidence interval in our population means of vertical axis by post-menstrual age (Table 5 and Figure 6).

**Table 5:** Mean US vertical axis (sd) in millimeters per week of post-menstrual age and 95% CI for population means.

**PMA (weeks)**	***N***	**Mean vertical axis (sd)(observed)**	**95% CI for population means**
24	8	51.21 (2.05)	49.50–52.93
25	12	53.53 (2.54)	51.92–55.14
26	17	56.66 (3.13)	55.05–58.27
27	15	58.94 (4.64)	56.37–61.51
28	16	59.71 (4.60)	57.25–62.16
29	25	62.92 (4.07)	61.25–64.60
30	39	64.98 (4.23)	63.61–66.36
31	55	66.32 (3.44)	65.39–67.25
32	62	67.76 (3.71)	66.81–68.70
33	47	70.09 (3.46)	69.07–71.10
34	34	70.48 (3.14)	69.39–71.58
35	30	73.77 (3.78)	72.36–75.18
36	24	74.23 (4.57)	72.30–76.16
37	15	75.03 (4.19)	72.71–77.35
38	11	78.82 (3.43)	76.52–81.13
39	8	79.58 (4.37)	75.93–83.23
40	8	78.97 (7.12)	73.01–84.93

### Prediction of TBV Through Linear Axis by 3D US and MRI

Once we obtained good reliability of TBV measured by 3D US and MRI and excellent agreement regarding the linear measurements that represent the brain axes according to the three orthogonal planes, we wanted to take a step further to facilitate the TBV estimation from 2D US. To do this we made a predictive model of TBV based on the biparietal diameter, the anteroposterior axis, and the vertical axis. We made a model for US and another one for MRI and compared both models.

### US TBV Prediction Based on US Linear Measurements

The relation between the brain axes and TBV was estimated by multilevel analysis, adjusting for repeated measurements. A detailed description of the estimated parameters can be seen in Table 6. The estimated TBV based on the axes would follow this equation:

$$\begin{array}{l} US\;TBV=-390.9+2.5^{*}BPD+3.4^{*}Vertical\;axis+2.3^{*}AP\;axis \end{array}$$

**Table 6:** Parameters of US TBV prediction mixed effect model by three orthogonal axes (BPD, anteroposterior, and vertical axes).

**US TBV**	**Coef**.	**Std. Err**.	***z***	***P* > |*z*|**	**[95% Conf. Interval]**	**Random-effects parameters**			
							**Estimate**	**Std. Err**.	**[95% Conf. interval]**
BPD	2.5	0.219	11.41	0.0001	2.070–2.929	var(_cons)	129.014	28.256	83.987–198.181
Vertical axis	3.420	0.239	14.30	0.0001	2.951–3.889	Var (Residual)	153.525	12.073	131.596–179.109
AP axis	2.324	0.148	15.65	0.0001	2.033–2.615	Number of obs = 422; number of groups = 87.			
_cons	−390.87	9.504	−41.13	0.0001	−409.495 to −372.240	Log likelihood = −1726; Wald chi2(3) = 5841.72; *p* = 0.0001			

### MRI TBV Prediction Based on MRI Linear Measurements

In a similar manner the relation between the brain axes and the MRI-TBV segmentation was estimated by multilevel analysis, adjusting for repeated measurements. A detailed description of the estimated parameters can be seen in Table 7. The estimated MRI-TBV based on the axes would follow this equation:

$$MRI\;TBV=-520.7+3.1^{*}BPD+4.2^{*}Vertical\;axis+2.7^{*}AP\;axis$$

**Table 7:** Parameters of MRI TBV prediction mixed effect model by three orthogonal axes (BPD, anteroposterior, and vertical axis).

**MRI TBV**	**Coef**.	**Std. Err**.	***z***	***P* > |*z*|**	**[95% Conf. Interval]**	**Random-effects parameters**			
							**Estimate**	**Std. Err**.	**[95% Conf. interval]**
BPD	3.075	0.833	3.69	0.0001	1.441–4.707	var(_cons)	1.20e+05	2.06e+05	4214.7–3.43e+06
Vertical axis	4.239	1.045	4.06	0.0001	2.191–6.287	Var (Residual)	1.19e+06	2.61e+05	7.75e+05–1.83e+06
AP axis	2.668	0.773	3.45	0.001	1.153–4.184	Number of obs = 92; number of groups = 62			
_cons	−520.7	25.78	−20.20	0.0001	−571.301 to −470.232	Log likelihood = −1096.12; Wald chi2(2) = 1024.55; *p* = 0.0001			

### Comparison of TBV Predicted by Linear Axis Through US vs. MRI

**Figure 7:**
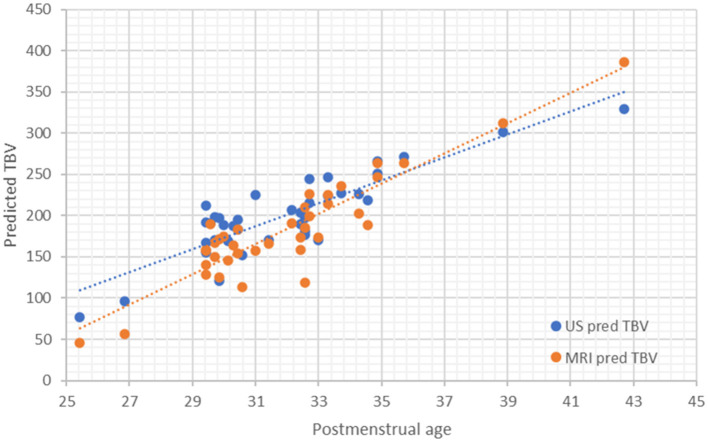
Scatterplot and correlation of predicted-by-axis-TBV by axis in MRI and US.

Predicted-by-axis-TBV estimated in US is reliable compared to the estimated by MRI with a non-significant mean difference of 17.81 cm^3^ (Pearson *r* = 0.983; *P* = 0.09) (Figure 7).

Once we showed that we could accurately predict TBV in US by measuring the three proposed axes we wanted to adapt an easy-to-use table that would further enable TBV use in routine clinical practice. The proposed model detailed in Table 6 and summarized in the equation: US TBV = −390.9 + 2.5^*^BPD + 3.4^*^Vertical axis + 2.3^*^AP axis; was tested for external validation showing a loss of prediction (shrinkage) of 2.3%. These results suggest its reliability as shrinkage is <10%. Moreover, we obtained a *R*${}_{\text{Mean}}^{2}$ = 0.927 on the internal validation test, which proves the model to have high predictive ability when performed on external data was. We then estimated the predicted TBV based on the three axis and summarized it in an easy-to-use table (see Table 8).

**Table 8:** Table of predicted US TBV in cm^3^ based on BPD (mm), anteroposterior axis (mm), and vertical axis (mm).

**BPD (mm)**	**Vertical axis (mm)**	**Anteroposterior axis (mm)**						
		**60**	**70**	**80**	**90**	**100**	**110**	**120**
40	45					95.4	118.7	141.9
	55			83.2	106.4	129.6	152.9	176.1
	65	70.9	94.1	117.4	140.6	163.9	187.1	210.3
	75	105.1	128.3	151.6	174.8	198.1	221.3	244.5
	85	139.3	162.5	185.8	209.0	232.3	255.5	278.7
	95	173.5	196.7	220.0	243.2	266.5	289.7	312.9
45	45				84.7	107.9	131.2	154.4
	55			95.7	118.9	142.1	165.4	188.6
	65	83.4	106.6	129.9	153.1	176.4	199.6	222.8
	75	117.6	140.8	164.1	187.3	210.6	233.8	257.0
	85	151.8	175.0	198.3	221.5	244.8	268.0	291.2
	95	186.0	209.2	232.5	255.7	279.0	302.2	325.4
50	45			74.0	97.2	120.4	143.7	166.9
	55		84.9	108.2	131.4	154.6	177.9	201.1
	65	95.9	119.1	142.4	165.6	188.8	212.1	235.3
	75	130.1	153.3	176.6	199.8	223.1	246.3	269.5
	85	164.3	187.5	210.8	234.0	257.3	280.5	303.7
	95	198.5	221.7	245.0	268.2	291.5	314.7	337.9
55	45			86.5	109.7	132.9	156.2	179.4
	55	74.2	97.4	120.7	143.9	167.1	190.4	213.6
	65	108.4	131.6	154.9	178.1	201.3	224.6	247.8
	75	142.6	165.8	189.1	212.3	235.6	258.8	282.0
	85	176.8	200.0	223.3	246.5	269.8	293.0	316.2
	95	211.0	234.2	257.5	280.7	304.0	327.2	350.4
60	45			99.0	122.2	145.4	168.7	191.9
	55	86.7	109.9	133.2	156.4	179.6	202.9	226.1
	65	120.9	144.1	167.4	190.6	213.8	237.1	260.3
	75	155.1	178.3	201.6	224.8	248.0	271.3	294.5
	85	189.3	212.5	235.8	259.0	282.3	305.5	328.7
	95	223.5	246.7	270.0	293.2	316.5	339.7	362.9
65	45		88.2	111.5	134.7	157.9	181.2	204.4
	55	99.2	122.4	145.7	168.9	192.1	215.4	238.6
	65	133.4	156.6	179.9	203.1	226.3	249.6	272.8
	75	167.6	190.8	214.1	237.3	260.5	283.8	307.0
	85	201.8	225.0	248.3	271.5	294.8	318.0	341.2
	95	236.0	259.2	282.5	305.7	329.0	352.2	375.4
70	45	77.5	100.7	124.0	147.2	170.4	193.7	216.9
	55	111.7	134.9	158.2	181.4	204.6	227.9	251.1
	65	145.9	169.1	192.4	215.6	238.8	262.1	285.3
	75	180.1	203.3	226.6	249.8	273.0	296.3	319.5
	85	214.3	237.5	260.8	284.0	307.3	330.5	353.7
	95	248.5	271.7	295.0	318.2	341.5	364.7	387.9
75	45	90.0	113.2	136.5	159.7	182.9	206.2	229.4
	55	124.2	147.4	170.7	193.9	217.1	240.4	263.6
	65	158.4	181.6	204.9	228.1	251.3	274.6	297.8
	75	192.6	215.8	239.1	262.3	285.5	308.8	332.0
	85	226.8	250.0	273.3	296.5	319.7	343.0	366.2
	95	261.0	284.2	307.5	330.7	354.0	377.2	400.4
80	45	102.5	125.7	149.0	172.2	195.4	218.7	241.9
	55	136.7	159.9	183.2	206.4	229.6	252.9	276.1
	65	170.9	194.1	217.4	240.6	263.8	287.1	310.3
	75	205.1	228.3	251.6	274.8	298.0	321.3	344.5
	85	239.3	262.5	285.8	309.0	332.2	355.5	378.7
	95	273.5	296.7	320.0	343.2	366.5	389.7	412.9
85	45	115.0	138.2	161.5	184.7	207.9	231.2	254.4
	55	149.2	172.4	195.7	218.9	242.1	265.4	288.6
	65	183.4	206.6	229.9	253.1	276.3	299.6	322.8
	75	217.6	240.8	264.1	287.3	310.5	333.8	357.0
	85	251.8	275.0	298.3	321.5	344.7	368.0	391.2
	95	286.0	309.2	332.5	355.7	378.9	402.2	425.4

An interval of 5 mm distance for BPD whereas for anteroposterior axis and vertical axis a 10 mm distance interval was chosen. Based on the model previously described where US TBV= −390.9 + 2.5^*^BPD +3.4^*^Vertical axis+ 2.3^*^AP axis.

## Discussion

Our study shows that monitoring brain growth in preterm infants during early life is feasible through TBV estimation based on 2D US measurement of the three orthogonal axes (BPD, vertical axis, and anteroposterior axis). Linear brain measurements of BPD, vertical axis, and anteroposterior axis are reliable when measured through US and show good agreement with MRI measures.

We found, as other authors had previously reported (6) excellent intra- and interobserver agreement for parenchymal BPD. Interestingly, the intra- and interobserver agreement of the anteroposterior and vertical axis is almost perfect in our study. While our results for anteroposterior axis are consistent with the results obtained in other studies (6, 21), we propose a systematic approach ensuring a 90 degrees angle among the anteroposterior axis and the vertical axis. Moreover, the proposed vertical axis takes into account the orientation of the sagittal view and the vermis anatomical landmarks, not the foramen magnum as suggested by Graça et al. (12) as we did not want to add the distance of the cisterna magna to a measure of brain growth, given its physiologic variability. This vertical axis could be suggested as more feasible in terms of its widespread use yet ensuring accuracy and reproducibility (6, 9, 22).

3D US enables obtaining optimal two-dimensional images of the desired anatomical section in all the explorations performed, while previous 2D ultrasound studies have found optimal planes obtained in 67% of the cases (23). This is the first time, to our knowledge, that the inter- and intraobserver agreement of these linear brain measurements has been studied using 3D US. Remarkably we achieved an overall better intra-observer agreement which could be attributable to the optimization of anatomical section selection achieved with the use of 3D US. Nevertheless, training and expertise in 3D US could have also contributed as our group has been working with 3D US for the last decade.

TBV measured by MRI has been associated with different perinatal morbidities (24) and neurodevelopment (25). We have studied the concordance between TBV measured by US 3D and MRI and found an excellent agreement between both methods (ICC of 0.98). Moreover, we have proven that TBV based on the three orthogonal axes is reliable for both US and MRI. Through this we have proved that an accurate estimation of TBV can be achieved through three very simple linear measures and we believe it could lead to a change in the clinical practice since TBV estimation could be introduced as a tool to monitor brain growth of the preterm infant during admission. We propose an easy to use equation and a table of predicted TBV that would enable implementing TBV in routine neonatal care.

The achieved sample size of patients and neuroimaging techniques has allowed us to describe the observed mean and the 95% confidence interval for population means which could be of further interest in the neonatal brain growth assessment. As 3D US is not widely available we have focused on its utility to make 2D US measurements reliable compared to a 3D US manual segmentation and to both linear and volumetric MRI measures. However, this study reinforces the potential role 3D US has in the NICU which has been previously recognized by other authors (26, 27) and by our group (14, 16, 28, 29). 3D allows navigation through the three planes once a whole brain acquisition has been obtained, is faster than 2D US and allows review offline of any possible section of interest instead of having a static 2D image saved (30–32). We suggest that 3D US routine implementation in the NICU could lead to a whole new approach to the central nervous system in the neonatal period, with a better evaluation of brain growth, maturation, and brain injury (32–34).

Our study is subject to several limitations that need to be considered. We studied images of VLBWI without brain damage and the anatomic landmarks of these measurements may not be clear in the presence of brain injury; the results should therefore also be validated in a cohort with brain damage. Moreover, only 38 patients (24.3%) were classified as having brain injury, which is a smaller number than expected in a VLBW population and it is therefore possible that we did include infants with the mildest forms of brain injury (both GMH-IVH and white matter injury). However, our study intended to establish the relationship of linear measurements with TBV and cannot be taken as a measure of brain growth. A more detailed study on all known factors that compromise brain growth (comorbidities, sex, gestational age) in a preterm population that includes those with brain injury was beyond the scope of this study but is warranted in the near future by our group. This might help to reach a better understanding of normal brain growth pattern vs. a hypothesized deviated pattern in the sickest preterm infant. We must also acknowledge that, although we used the MRI slices that were closest to the ultrasound images, we are measuring structures acquired with different angulations: US images are obtained in coronal and sagittal planes through the anterior fontanel while MRI axial and coronal planes are used with coronal planes being parallel. Nonetheless, the measurements proposed should not be affected by the angulation difference; moreover, in our study, the use of 3D US has allowed us to select offline those anatomical sections that most closely resemble those obtained with MRI. As this study was performed in preterm infants this methodology would need to be assessed separately in term infants.

In conclusion, we have found that US measurements of BPD, vertical axis and anteroposterior axis are reliable. TBV segmented through 3D US is reliable and accurate compared to MRI measured TBV. When 3D US is not available, 2D US TBV estimation could be achieved through biparietal diameter, vertical and anteroposterior axis which could lead to a better assessment of brain growth in the preterm infant and could potentially be added to routine 2D US in the NICU.

## Data Availability Statement

The raw data supporting the conclusions of this article will be made available by the authors, without undue reservation.

## Ethics Statement

The studies involving human participants were reviewed and approved by Research and Ethics Committee, Cádiz. Written informed consent to participate in this study was provided by the participants' legal guardian/next of kin.

## Author Contributions

IB-F and SL-L conceptualized and designed the study, participated in recruitment and performed the measurements, supervised data collection and performed analysis, drafted the initial manuscript, reviewed the manuscript, and approved the final manuscript as submitted. ER-G, ML-G, SL-F, CR-C, PO-D, and YC contributed to data collection and performed the measurements, reviewed the manuscript, and approved the final manuscript as submitted. All authors approved the final manuscript as submitted and agree to be accountable for all aspects of the work.

## Conflict of Interest

The authors declare that the research was conducted in the absence of any commercial or financial relationships that could be construed as a potential conflict of interest.
